# Association of Lecithin Cholesterol Acyltransferase rs5923 Polymorphism in Iranian Individuals with Extremely Low High-Density Lipoprotein Cholesterol: Tehran Lipid and Glucose Study

**DOI:** 10.7508/ibj.2015.03.007

**Published:** 2015-07

**Authors:** Mohsen Naseri, Mehdi Hedayati, Maryam Sadat Daneshpour, Fatemeh Bandarian, Fereidoun Azizi

**Affiliations:** 1 *Genomic Research Center, Birjand university of Medical Sciences, Birjand, Iran; *; 2 *Cellular and Molecular Endocrine Research Center, Research Institute for Endocrine Sciences, Shahid Beheshti University of Medical Sciences, Tehran, Iran;*; 3 *Diabetes Research Center, Endocrinology and Metabolism Clinical Sciences Institute, Tehran University of Medical Sciences, Tehran, Iran*

**Keywords:** Polymorphism, Single nucleotide, Lipoproteins

## Abstract

**Background:**

The serum concentration of high-density lipoprotein cholesterol (HDL-C) is one of the important heritable risk factors for cardiovascular disease and is a target for therapeutic intervention. In this study, we aimed to evaluate the effects of lecithin cholesterol acyltransferase (*LCAT*) gene polymorphism rs5923 on LCAT enzyme activity and serum HDL-C concentration.

**Methods:**

The study population was selected from consecutive individuals with HDL-C ≤ 5^th^ percentile (n = 73) and extremely high HDL-C ≥ 95^th^ percentile (n = 57) who had participated in the Tehran Lipid and Glucose Study. The rs5923 polymorphism was genotyped using direct sequencing. LCAT activity was measured by fluorometric assay kit, and lipid concentrations were measured using the enzymatic colorimetric method.

**Results:**

The genotype frequencies were significantly different between the high HDL-C group (CC 94.7%, CT 5.3%) and the low HDL-C group (CC 83.6%, CT 16.4%) (*P* = 0.048). The T-allele frequencies in subjects with low and high HDL-C were 0.082 and 0.026, respectively (*P* = 0.16). The association of the single-nucleotide polymorphism rs5923 with low HDL-C was not statistically significant after adjustment for age, sex, and BMI (odd ratio = 2.65, 95% *confidence interval* = 0.32-21.5, *P* = 0.36, regression logistic analysis). Also, the effects of LCAT enzyme activity did not depend on the HDL-C level (*P* = 0.24).

**Conclusion:**

rs5923 polymorphism is not associated with low HDL-C levels in Iranian population.

## Introduction

The serum concentration of high-density lipoprotein cholesterol (HDL-C) is one of the important heritable risk factors for cardiovascular disease (CVD), and it is a target for therapeutic intervention [1]. Although high plasma levels of HDL-C are protective against CVD, a low plasma level of HDL-C (<40 mg/ dl) is a strong and an independent risk factor for development of premature atherosclerosis leading to CVD [1, 2]. In individuals participated in the Tehran Lipid and Glucose Study (TLGS), the prevalence of cardiovascular risk factors, particularly low HDL-C levels was high (32%) among the urban population of Tehran [3]. 

 There is overwhelming evidence that in addition to environmental factors such as decreased exercise, alcohol consumption, cigarette smoking, diabetes, dietary saturated fat, and obesity, the genetic background also plays an important role in serum HDL-C levels [4]. The heritability of HDL has been estimated to be in a range of 40-60%. Moreover, in recent decades, various studies have been conducted to investigate gene regulation [5]. 

 Molecular defects in gene apolipoprotein A-I, adenosine triphosphate binding cassette transporter A1, and lecithin cholesterol acyltransferase (*LCAT*) may lead to rare genetic forms of HDL deficiency [6]. LCAT, first described in 1962 [7], is a soluble enzyme that plays a central role in the formation and maturation of HDL. It is a catalyst for transferring acyl groups from C2 position of lecithin to the 3-hydroxy group of cholesterol that produces most cholesterol ester in the plasma [8]. This enzyme converts cholesterol and lecithins to cholesteryl esters and lysophosphatidylcholines on the HDL surface [9]. The human *LCAT* gene, localized at 16q22, spans 4.2 kb and contains six exons, including ∼1.5-kb coding sequence that encodes a 416-amino-acid protein. LCAT is synthesized by the liver and circulates in blood plasma as a HDL composite. In the reverse cholesterol transport system, HDL particles receive the cholesterol of peripheral cells and carry them to the liver [9]. LCAT has also a significant role in the conversion of preβ HDL into mature spherical α-HDL and metabolism of intravascular HDL [10]. 

 The single-case investigations or studies of small families have led to the identification of most mutations in *LCAT* gene [8-10]. Two syndromes of LCAT deficiency are fish-eye disease (OMIM no. 136120) showing partial enzymatic defect and familial LCAT deficiency (OMIM no. 245900) in which enzyme activity is completely absent. Low plasma levels of HDL-C, apolipoprotein A-I and A-II levels as well as hypoalphalipoproteinemia and cholesterol esterification impairment are observed in individuals with fish-eye disease and familial LCAT deficiency [10]. So far, more than 80 causative mutations in LCAT have been described in the Human Gene Mutation Database [11]. 

 Furthermore, in recent years, 16 loci associated with HDL-C (*P *< 5 × 10^-8^) levels have been identified in the meta-analysis of a genome-wide study on over 100,000 individuals [12], showing that the strongest indicator of isolated variation in HDL-C levels is a single nucleotide polymorphism (SNP) in the *LCAT* gene [13]. More than 160 SNPs have been currently found in *LCAT* and submitted to the Single Nucleotide Polymorphism Database [14]. rs5923 is one of these SNP initially reported as 4886C>T(L369L) mutation in the human *LCAT* gene in 2000 [15]. In 2011, Agirbasli and co-workers [16] reported that this mutation is the most frequent *LCAT* gene variant in the Turkish population, and T-allele frequency was predominant in individuals with low HDL-C. In the present investigation, we aimed to evaluate the effects of *LCAT* gene polymorphism rs5923 on LCAT enzyme activity and HDL-C level in an Iranian population.

## MATERIALS AND METHODS

 This cross-sectional study was conducted on participants enrolled in the TLGS, which is a prospective population-based, longitudinal cohort study with more than 15,000 participants and designed for the assessment of cardiovascular risk factors and diseases in the urban residents of Tehran [17]. 

 Individuals aged 15-70 years with HDL-C levels of < the 5^th^ percentile for age and sex (n = 73) and those with >95^th^ percentile (n = 57) for age and sex were selected from the TLGS population. Individuals who had the same trait in 4 TLGS phases persistently and had at least a family member with the same trait were included in the study. Also, obese individuals (BMI ≥ 30 kg/m^2^) and those receiving drugs affecting HDL-C levels were excluded from the study.

 An informed consent was obtained from the participants and ethics approval was given by the Ethics Committee of the Research Institute for Endocrine Sciences at Shahid Beheshti University of Medical Sciences, Tehran, Iran. A standardized questionnaire was used to gather data on medication usage, demographics, sex, age, lipid disorders, and hypertension treatment. Details of the collection, preparation, and lipid determination methods together with details on quality control have been described previously [18]. 

The activity of plasma LCAT was measured quantitatively via a fluorometric assay kit (Calbio-chem, Germany). Briefly, fluorescence was emitted from the florescent substrate at 470 nm. Human plasma samples and substrates were incubated at 37ºC for 18 hours. Upon hydrolyzation of the substrate, a monomer was discharged by LCAT producing 390-nm fluorescence. Change in the intensity of 470/390 emission was used to assess LCAT activity.

For assessment of the *LCAT* polymorphism, Buffy coats separated from the non-coagulated whole blood samples were stored at -70°C before processing. The genomic DNA was extracted using salting out method [17]. The sequences of human *LCAT* coding region were obtained from the genome browser UCSC (http://genome.ucse.edu) [19]. Primers were designed to amplify coding sequence and exon-intron boundaries of the *LCAT* (NM_000229.1) using web-based Primer3 software ( version 0.4.0) and NCBI primer-blast programs [20]. 

Hybridization was carried out in a DNA thermal cycler (Corbett, Australia). The genomic region flanking the rs5923 (accession no. NT_010498.15 :g.21588152G>A) polymorphism in exon 6 of *LCAT* was amplified with forward (5'-TGAGCCTACACTC AGCAGGTTGTG -3') and reverse (5'-CCCATCTT GCCTCACTGCACACA-3') primers using the following thermal cycles: initial denaturation at 96°C for 8 min, followed by 32 cycles of denaturation (96°C/1min), annealing (69.5°C/1 min), and extension (72°C/1 min) with a final extension at 72°C for 7 min.

Fisher’s exact test was used to assess deviation from Hardy-Weinberg equilibrium. The distribution of categorical variables was examined using the Chi- square test. Logistic regression analysis estimated the genotypic OR of *LCAT* polymorphism for each categorical variable, including combined effects of age and sex. Student’s *t* test was used to assess differences in *LCAT* mutant activity in comparison to the wild-type *LCAT*. SPSS (version 15.0; SPSS, Chicago, IL, USA) was used for data analysis. *P* ≤ 0.05 was deemed statistically signiﬁcant. 

**Table 1:** Characteristics of the study population

**Variables**	**Subjects with high ** **HDL-C (n = 57)**	**Subjects with low ** **HDL-C (n = 73)**	***P*** ** value**
Sex (M/F) (%)	38/56	61/43	NS
Age (yr)	37 ± 16	41 ± 13	NS
TC (mg/dl)	194 ± 42	168 ± 47	0.001
LDL-C (mg/dl)	104 ± 35	90 ± 28	0.012
TG (mg/dl)	81 ± 42	251 ± 179	0.000
BMI (kg/m^2^)	23 ± 3	26 ± 3	0.001
Glucose (mg/dl)	93 ± 9	108 ± 34	0.001
LCAT activity (nmol/ml/h)	103 ± 14	106 ± 15	NS
SBP (mmHg)	113 ± 19	114 ± 17	NS
DBP (mmHg)	73 ± 11	77 ± 9	0.016

BMI, body mass index; HDL-C, high-density lipoprotein cholesterol; LCAT, lecithin:cholesterol acyltrans- ferase; LDL-L, low-density lipoprotein; TC, total cholesterol; TG, triglycerides; DBP, diastolic blood pressure; SBP, systolic blood pressure. *NS* not significant. Demographics and lipid levels among subjects with high and low HDL-C levels are shown

## Results

The demographic data, clinical status, and biochemical parameters of the studied population are presented in Table 1. As shown in the Table, the mean ± SD age of the extremely high HDL-C group was 37 ± 16 years and that of the extremely low HDL-C group was 41 ± 13 years. BMI, fasting blood sugar, cholesterol, triglyceride, and LDL-C in extremely high HDL-C group were signiﬁcantly different from the extremely low HDL-C group, whereas LCAT activity did not differ significantly.

 Rs5923 is a synonymous variation existing in the exon 6 of *LCAT* gene. The genotype frequencies of *LCAT* gene (rs5923) polymorphism in individuals with high and low HDL-C levels are shown in Table 2. The rs5923 polymorphism genotype frequencies were in accordance with Hardy-Weinberg Equilibrium (K^2^ = 0.78). The genotype (C/C:C/T genotypes) distributions of rs5923 in the high HDL and low HDL-C groups were 94.7%:5.3% and 83.5%: 16.5%, respectively. No individual was homozygote for the TT genotype. The genotype distribution of the SNP was significant between individuals with high and low HDL levels (*P* = 0.048, Table 2). The allele (C:T) distributions of rs5923 in the high and low HDL-C groups were 91.7%: 8.3% and 97.3%: 0.27%, respectively (*P* = 0.16). However, the presence of the T allele did not increase the risk of having a lower HDL level as compared to the C allele (odd ratio [OR] = 3.54, confidence interval [CI]: 0.9-13.21; *P* = 0.06). The results suggest that rs5923 of *LCAT* may not contribute to the risk for low HDL susceptibility. The mean values of lipid levels, fasting blood sugar, and LCAT activity among genotypes are shown in Table 3. Among the subjects with low HDL and those with high HDL, no significant differences were observed in the level of clinical factors among the rs5923 genotypes.

## Discussion

In the 1980s, low HDL-C levels in serum were identified as a risk factor for coronary artery disease [21]. The low level of HDL-C are a risk factor for metabolic syndrome, which is a disorder with the following medical conditions: abdominal (central) obesity, elevated blood pressure, elevated fasting plasma glucose, high serum triglycerides, and low HDL levels, which increase the risk for development of CVD [22].

Low levels of HDL-C are common among Iranian population [17]. HDL compared to the other lipoproteins (i.e., very-low-density lipoprotein, *intermediate-density lipoprotein, *and chylomicrons) is more tightly controlled by genetic factors. For example, in some families with Japanese ancestry, a genetic deficiency of cholesteryl ester transfer protein is significantly associated with elevated HDL-C levels [23]. In the current study, we investigated the correlation between a single polymorphism nucleotide of *LCAT* and HDL-C levels. No association was found between the genotypes for the rs5923 polymorphism of the *LCAT* gene and HDL-C levels. Also, no significant correlation was found between LCAT enzyme activity and lipid levels. 

**Table 2:** Genotype frequencies of rs5923 SNP in HDL-C groups

**Group **	**C/C**	**C/T**
Low HDL-C (%)	83.6	16.4
High HDL-C (%)	94.7	5.3

HDL-C, high-density lipoprotein cholesterol; LCAT, lecithin: cholesterol acyltransferase. Genotype frequencies of SNP 4886C/T in the *LCAT* gene among subjects with low and high HDL-C levels (*P* = 0.049)

**Table 3:** Mean values of lipids levels, FBS, and LCAT activity among genotypes

**rs5923** ** polymorphism**	**Total**				**Low HDL**				**High HDL**		
	**CC**	**CT**	***P*** ** value**		**CC**	**CT**	***P*** ** value**		**CC**	**CT**	***P *** **value**
HDL-C levels (mg/dl)	50 ± 24	35 ± 18	0.02		28 ± 3	26 ± 2	0.10		74 ± 10	70 ± 4	0.41
TG levels (mg/dl)	171 ± 166	239 ±182	0.14		106 ± 15	105 ± 21	0.76		81 ± 44	54 ± 12	0.30
LDL levels (mg/dl)	98 ± 32	99 ± 36	0.93		250 ± 192	285 ± 175	0.56		106 ± 35	97 ± 49	0.69
TC levels (mg/dl)	183 ± 48	173 ± 43	0.46		91 ± 27	100 ± 35	0.38		197 ± 43	178 ± 45	0.46
FBS (mg/dl)	99 ± 21	111 ± 48	0.08		170 ± 49	172 ± 45	0.91		92 ± 8	83 ± 4	0.08
BMI (kg/m^2^)	25 ± 3	26 ± 3	0.13		105 ± 27	118 ± 52	0.18		23 ± 3	24 ± 3	0.67
LCAT activity (nmol /ml/h)	105 ± 14	103 ± 19	0.80		26 ± 3	27 ± 2	0.48		103 ± 13	98 ± 12	0.57

HDL-C, high-density lipoprotein cholesterol; LCAT, lecithin: cholesterol acyltransferase; LDL-L, low-density lipoprotein; TC, total cholesterol; TG, triglycerides ; FBS, fasting blood glucose. Distribution of mean values with standard deviations of HDL-C levels, LCAT activity among LCAT genotypes

*LCAT* gene is polymorphous in Iranian population, and some of its common variants are the ones previously reported in other Turkish, European, and Canadian societies. In a meta-analysis study performed on 20,562 individuals in Denmark, it has been shown that 1,045 people had rs5923 polymorphism (T-allele frequency: 0.05) [13], which is compatible with our results. In this investigation, the frequency of mutant T allele in the *LCAT* rs5923 polymorphism was 0.058. Also, this finding was consistent with the results of Recalde and co-workers [24] who found that the T-allele frequency of the rs5923 polymorphism was 0.07 in Spanish individuals with hypoalphalipoproteinemia. This frequency is considered as being high in the Turkish population (0.54) [16]. However, according to the reports of NHLBI GO Exome Sequencing Project on 4,348 individuals, the T-allele frequency of the rs5923 polymorphism was 0.098. Furthermore, the T-allele frequency in rs5923 demonstrated a considerable ethnic divergence: 0.212 in the Sub-Saharan African inhabitants, 0.035 in the European population, 0.09 in the African American, while being 0.03 in the Asian population [14]. 

In the initial analysis of the data, rs5923 showed a significantly higher occurrence in the low HDL than the high HDL levels. However, no significant association was observed among the low HDL levels after analyzing the logistic regression and entering the confounding factors. Since the change in the base of this SNP does not lead to the change in amino acid, non-different presence of rs5923 in both groups is justifiable. Nevertheless, in a study conducted on 100 Turkish citizens, T-allele frequencies of this SNP were obtained 0.54 and 0.37 in the low and high HDL-C groups, respectively (*P* = 0.019) [16]. In another study, the frequencies of the T allele were different between low HDL-C individuals (0.064 and 0.059) and the control groups (0.035 and 0.081) [22]. A plausible reason for this observation is the possible association between the T allele and another allele of the relevant locus (the causative factor on enzyme function) in those populations. Moreover, the proximity of this polymorphism to the coding region of enzyme active site can somehow affect the enzyme activity in that community. Interestingly, in a study conducted by Ashley-Koch *et al.* [25], this SNP showed a significant relationship with the phenotype of pulmonary hypertension in the patients suffering from sickle cell disease. However, this observation has not been confirmed in any other studies. 

There are several limitations within the present study. The small number of our samples only covers individuals with no history of statin consumption. The reaction to statins and additional new therapies in order to increase the HDL-C levels could be modulated by LCAT enzyme. Quality rather than quantity of HDL-C provides more information regarding the HDL-C preventive role in cardiovascular effects. 

The T-allele frequencies of *LCAT* rs5923 polymorphism were not significantly different in subjects with low and high HDL-C. The fact that there is no association between rs5923 polymorphism and low HDL-C levels probably shows that it is not an important risk factor for HDL-C levels and consequently for CVD. 
